# Synthesis, Anticonvulsant, Sedative and Anxiolytic Activities of Novel Annulated Pyrrolo[1,4]benzodiazepines

**DOI:** 10.3390/ijms150916500

**Published:** 2014-09-18

**Authors:** Kumaraswamy Sorra, Chien-Shu Chen, Chi-Fen Chang, Srinivas Pusuluri, Khagga Mukkanti, Chi-Rei Wu, Ta-Hsien Chuang

**Affiliations:** 1 Medicinal Chemistry Laboratory, Gunapati Venkata Krishnareddy Biosciences Private Limited, Plot No. 28A, Industrial Development Area Nacharam, Hyderabad 500076, India; E-Mails: kumaraswamy.s@gvkbio.com (K.S.); pusulurisrinivas@gvkbio.com (S.P.); 2 Chemistry Division, Institute of Science and Technology, Jawaharlal Nehru Technological University, Kukatpally, Hyderabad 500085, India; E-Mail: kmukkanti@yahoo.com; 3 School of Pharmacy, China Medical University, Taichung 40402, Taiwan; E-Mail: cschen7@mail.cmu.edu.tw; 4 Department of Anatomy, School of Medicine, China Medical University, Taichung 40402, Taiwan; E-Mail: cfchang@mail.cmu.edu.tw; 5 Department of Chinese Pharmaceutical Sciences and Chinese Medicine Resources, China Medical University, Taichung 40402, Taiwan; 6 Research Center for Chinese Herbal Medicine, China Medical University, Taichung 40402, Taiwan

**Keywords:** benzodiazepine, thiadiazolone, pyrimidinone, cyclocondensation, anticonvulsant, sedative, anxiolytic

## Abstract

Four new pentacyclic benzodiazepine derivatives (PBDTs **13**–**16**) were synthesized by conventional thermal heating and microwave-assisted intramolecular cyclocondensation. Their anticonvulsant, sedative and anxiolytic activities were evaluated by drug-induced convulsion models, a pentobarbital-induced hypnotic model and an elevated plus maze in mice. PBDT **13**, a triazolopyrrolo[2,1-*c*][1,4]benzodiazepin-8-one fused with a thiadiazolone ring, exhibited the best anticonvulsant, sedative and anxiolytic effects in our tests. There was no significant difference in potency between PBDT **13** and diazepam, and we proposed that the action mechanism of PBDT **13** could be similar to that of diazepam via benzodiazepine receptors.

## 1. Introduction

**Figure 1:**
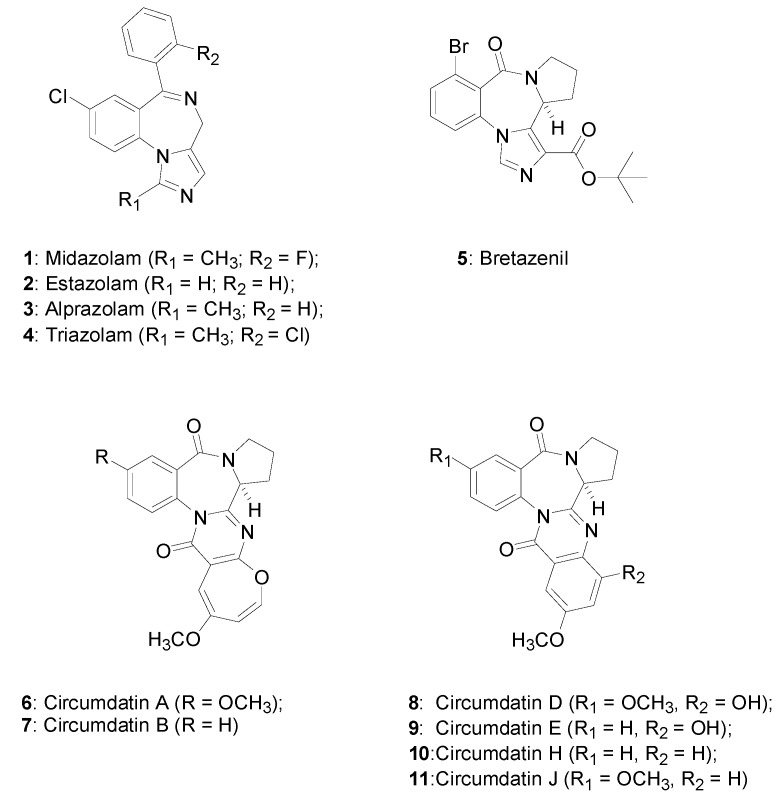
Members of the fused hetero-cyclic benzodiazepine family.

The benzodiazepine nucleus is a privileged scaffold that has emerged as a core structural fragment of various muscle relaxant, anxiolytic and anticonvulsant agents [1,2,3,4,5]. Tricyclic, tetracyclic and polycyclic benzodiazepines fused with various hetero-cyclic rings have been a focus of study in the field of medicinal chemistry. Midazolam (**1**), estazolam (**2**), alprazolam (**3**) and triazolam (**4**) are fused imidazole- and triazole-benzodiazepines, and these are well known as psychotropic agents (Figure 1) [6,7,8,9,10]. The tricyclic pyrrolobenzodiazepines (PBDs) are an important class of sequence-selective DNA interactive agents, and they are produced by *Streptomyces* species as natural antitumor antibiotics [11,12,13,14]. Bretazenil (**5**), a tetracyclic benzodiazepine, has attracted interest in the treatment of CNS disorders and neurodegenerative diseases [15,16]. The polycyclic benzodiazepine alkaloids isolated from *Aspergillus* and *Penicillium* sp. species, such as circumdatins (**6**–**11**), benzomalvins, asperlicins and sclerotigenin, exhibit promising bioactivities [17,18,19,20,21,22]. Moreover, (−)-circumdatin H is an inhibitor of the mammalian mitochondrial respiratory chain; benzomalvins A–C show potent inhibitory activity against substance P at the guinea pig, rat and human neurokinin NK1 receptor, and asperlicin is well known as a potent cholecystokinin antagonist [23,24,25,26].

Recently, we have communicated the synthesis of amido-substituted triazolopyrrolo[2,1-*c*][1,4]benzodiazepine (pentacyclic benzodiazepine derivative (PBDT)) derivatives and the evaluation of their cytotoxicity against Mahlavu cells [27]. Intrigued by the interesting biological activities and our ongoing interest in benzodiazepine-derived compounds, we became interested in the synthesis of some model compounds of polycyclic benzodiazepine alkaloids. In this paper, we report the synthesis, anticonvulsant, sedative and anxiolytic activity of annulated pyrrolobenzodiazepines, based on the anxiolytic drug (**5**).

## 2. Results and Discussion

### 2.1. Synthesis

**Scheme 1:**
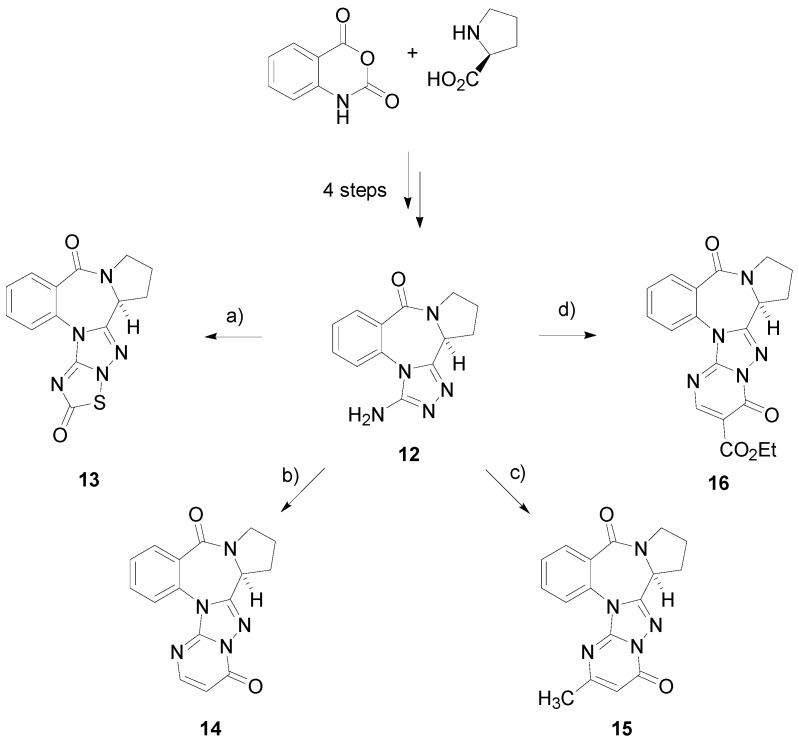
Synthesis of annulated benzodiazepines. Reagents and conditions: (**a**) chlorocarbonylsulfenyl chloride, Na_2_CO_3_, CH_2_Cl_2_–H_2_O, 0 °C, 30 min, 65% yield; (**b**) ethyl propiolate, EtOH, 150 °C, 20 min, MW, 66% yield; (**c**) ethyl acetoacetate, AcOH, 150 °C, 20 min, MW, 72% yield; (**d**) diethyl ethoxymethylenemalonate, EtOH, 150 °C, 20 min, MW, 61% yield.

The synthesis strategy for constructing the annulated pyrrolo[1,4]benzodiazepines derivatives **13**–**16** is shown in Scheme 1. 3-Amino triazolopyrrolo[2,1-*c*][1,4]benzodiazepin-8-one (**12**), a key intermediate for the synthesis of polycyclic benzodiazepine derivatives, has been synthesized from isatoic anhydride and l-proline in four steps according to our previous reported method [27,28]. The final pentacyclic benzodiazepine derivatives **13**–**16** were prepared by the intramolecular cyclocondensation of the tetracyclic intermediate **12** with chlorocarbonylsulfenyl chloride, ethyl propiolate, ethyl acetoacetate and diethyl ethoxymethylenemalonate. Firstly, the fused thiadiazolone derivative **13** was produced in a 65% yield by cyclocondensation of the intermediate **12** with chlorocarbonylsulfenyl chloride under basic conditions [29]. However, the cyclocondensation of bis-nucleophilic intermediate **12** with ethyl propiolate using conventional thermal heating (EtOH, reflux, 20 h) gave Compound **14** in a poor yield (15%), with unreacted starting material as the predominant species. The intramolecular cyclocondensation was efficiently promoted by a microwave-assisted method, and a fused pyrimidinone derivative **14** was obtained in a satisfactory yield (66%) [30]. Similarly, tetracyclic Compound **12** was treated with ethyl acetoacetate and diethyl ethoxymethylenemalonate under microwave conditions, affording the fused substituted pyrimidinone derivatives, **15** and **16**, in 72% and 61% yields, respectively [31].

### 2.2. Biology

Benzodiazepines are common muscle relaxant, anxiolytic and anticonvulsant agents, but their side effects limit their clinical use. It is significant to develop modified benzodiazepines to minimize the side effects. Four PBDT derivatives **13**–**16** are synthesized from a core skeleton tetracyclic 3-amino triazolopyrrolo[2,1-*c*][1,4]benzodiazepin-8-one (**12**), and we evaluated their anticonvulsant, sedative and anxiolytic activities by drug-induced convulsion models, a pentobarbital-induced hypnotic model, and an elevated plus maze (EPM) in mice.

Firstly, we selected two drugs (picrotoxin (10 mg/kg, sc) or strychnine (2 mg/kg, ip)) to induce convulsion to evaluate the anticonvulsant activities of PBDTs **13**–**16** and used diazepam as a positive control (Table 1). In the picrotoxin-induced convulsion model, only PBDT **13** could prolong the duration of clonic–tonic convulsion induced by picrotoxin or strychnine (*p* < 0.001). However, PBDT **14** only prolonged the duration of strychnine-induced, but not picrotoxin-induced, clonic–tonic convulsion (*p* < 0.001). On the contrary, PBDTs **15** and **16** only prolonged the duration of clonic–tonic convulsion induced by picrotoxin, but not strychnine (*p* < 0.05). Diazepam at 1 mg/kg also prolonged the latency of myoclonic jerks and the duration of clonic–tonic convulsion induced by picrotoxin or strychnine (*p* < 0.01, *p* < 0.001). Therefore, we suggested that PBDT **13** among PBDT derivatives possesses better anticonvulsant effects, and its anticonvulsant mechanism could be similar to diazepam, which mainly acts at benzodiazepine receptors.

**Table 1:** The effects of pentacyclic benzodiazepine derivatives (PBDTs) (1 mg/kg, ip) or diazepam (1 mg/kg, ip) on picrotoxin- and strychnine-induced convulsion in mice.

Treatment	Dose (mg/kg)	Picrotoxin		Strychnine	
		Latency (s)	Duration (s)	Latency (s)	Duration (s)
Vehicle	–	294.4 ± 26.6	182.9 ± 14.3	304.6 ± 12.4	184.7 ± 14.9
PBDT **13**	1	377.0 ± 28.1 *	445.7 ± 29.2 ***	260.6 ± 23.3	266.8 ± 7.9 ***
PBDT **14**	1	297.5 ± 3.2	181.1 ± 36.3	357.5 ± 30.5	325.5 ± 4.6 ***
PBDT **15**	1	312.8 ± 11.2	395.8 ± 38.9 ***	304.6 ± 21.7	171.6 ± 6.1
PBDT **16**	1	313.0 ± 18.7	296.9 ± 35.6 *	313.0 ± 18.7	185.6 ± 2.0
Diazepam	1	440.5 ± 20.1 **	481.1 ± 18.7 ***	367.1 ± 31.0 *	459.7 ± 24.0 ***

Values are the mean ± SEM, *n* = 4 mice; * *p* < 0.05, ** *p* < 0.01, *** *p* < 0.001, compared with the vehicle group.

**Figure 2:**
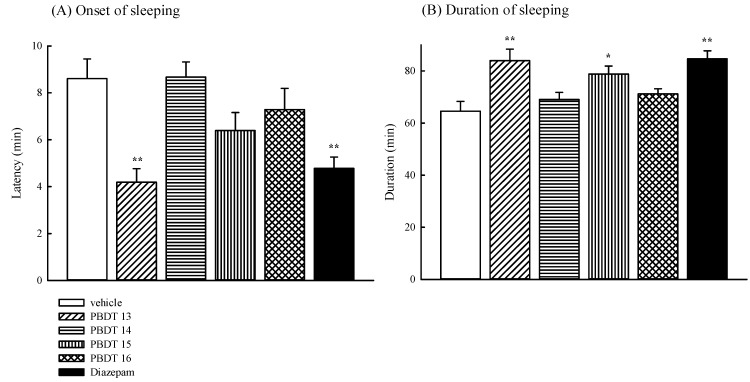
The effects of PBDTs **13**–**16** (1 mg/kg, ip) or diazepam (1 mg/kg, ip) on the (**A**) the latency to the loss of righting reflex and (**B**) total duration of sleeping time induced by sodium pentobarbital (30 mg/kg, ip). Values are the mean ± SEM, *n* = 4 mice; * *p* < 0.05, ** *p* < 0.01, compared with the vehicle group.

Next, we evaluated the sedative effects of PBDTs **13**–**16** by the pentobarbital-induced hypnotic model. The sedative effects of PBDTs **13**–**16** and diazepam on the pentobarbital (30 mg/kg, ip)-induced hypnotic model are shown in Figure 2. PBDTs **13** and **15** augmented the duration of sleeping time induced by pentobarbital (Figure 2B; * *p* < 0.05, ** *p* < 0.01), but only PBDT **13** shortened the onset of sleeping induced by pentobarbital (Figure 2A; ** *p* < 0.01). No significant changes in the onset of sleeping and the duration of sleeping time induced by pentobarbital were observed by the administration of PBDTs **14** and **16**. Diazepam at 1 mg/kg also induced a significant decrement in the onset of sleep and increased the duration of sleeping time (** *p* < 0.01). Therefore, we further suggested that only PBDT **13**, similar to diazepam, possesses better sedative effects via benzodiazepine receptors.

**Figure 3:**
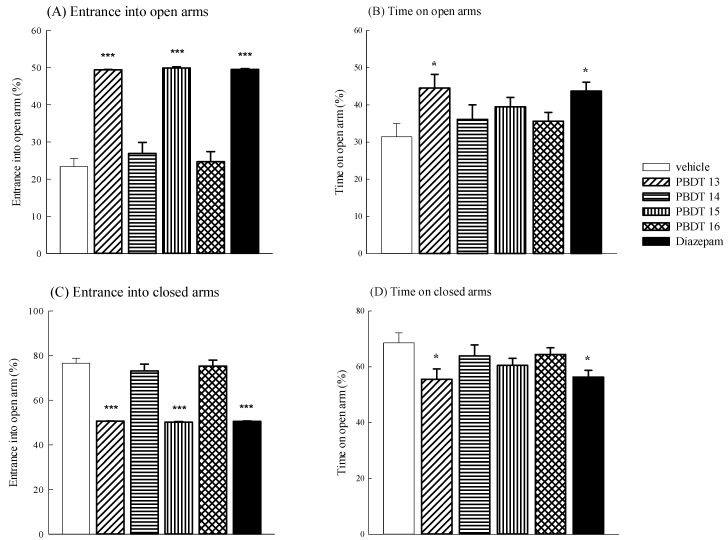
The effects of PBDTs **13**–**16** (1 mg/kg, ip) or diazepam (1 mg/kg, ip) on the (**A**) the percentage of open arm entries; (**B**) the percentage of time spent in open arm entries; (**C**) the percentage of closed arm entries and (**D**) the percentage of time spent in the closed arm entries of the elevated pus maze during a 5-min test in male mice. Values are the mean ± SEM, *n* = 4 mice; * *p* < 0.05, *** *p* < 0.001, compared with the vehicle group.

Finally, we evaluated the anxiolytic effects of PBDTs **13**–**16** by the elevated plus maze. EPM is the most popular test of anxiety and the first-choice test for screening anxiolytic drugs [32]. The anxiolytic effects of PBDTs **13**–**16** and diazepam on the elevated plus maze are shown in Figure 3. PBDTs **13** and **15** increased the percentage of the time spent in the open arms (*** *p* < 0.001), but only PBDT **13** increased the percentage of the entries into open arms in the elevated plus maze (* *p* < 0.05). No significant changes in the percentage of the entries into open arms and the time spent in the open arms in the elevated plus maze were observed by the administration of PBDTs **14** and **16**. Diazepam at 1 mg/kg also induced a significant increment in the percentage of the entries into open arms and the time spent in the open arms (* *p* < 0.05, *** *p* < 0.001). Therefore, we further suggested that only PBDT **13**, similar to diazepam, possesses a better anxiolytic effects via benzodiazepine receptors.

From these above results, we suggested that PBDT **13** possessed the best anticonvulsant, sedative and anxiolytic effects among PBDT derivatives **13**–**16**, and then, the next derivative was PBDT **15**. A new potential anxiolytic compound, PBDT **13**, was found. There was no significant difference in potency between PBDT **13** and diazepam in our tests, and the action mechanism of PBDT **13** was similar to that of diazepam via the benzodiazepine receptors.

## 3. Experimental Section

### 3.1. General

Melting points were recorded on a Yanaco MP-3 melting point apparatus (Yanaco Corp., Kyoto, Japan) and were not corrected. IR spectra were recorded on a Nicolet Magna FT-IR spectrophotometer (Nicolet Instrument, Inc., Madison, WI, USA). NMR spectra were recorded on Bruker AMX 500 FT-NMR spectrometers (Bruker, Karlsruhe, Germany); all chemical shifts were given in ppm from tetramethylsilane as an internal standard. Mass spectra were obtained on a VG 70-250S spectrometer by a direct inlet system (Micromass Corp., Manchester, UK).

(2*S*)-18-Thia-6,14,16,19,20-pentaazapentacyclo[12.6.0.0^2,6^.0^8,13^.0^15,19^] icosa-1(20),8,10,12,15-pentaene-7,17-dione (**13**): To a solution of Compound **12** (100 mg, 0.39 mmol) and Na_2_CO_3_ in CH_2_Cl_2_–H_2_O (0.5 mL, 1:1) was added chlorocarbonylsulfenyl chloride (51 mg, 0.39 mmol) slowly at 0 °C and stirred for 30 min at the same temperature. The reaction mixture was diluted with CH_2_Cl_2_, separating the organic layer. The aqueous layer was extracted with CH_2_Cl_2_. The combined organic layer was washed with brine solution, dried over Na_2_SO_4_ and concentrated. The residue was purified by column chromatography to afford **13** (80 mg, 65%) as a pale yellow solid. mp 284–286 °C; ν_max_ (KBr) 2850, 1705, 1627, 1573, 1465, 1411, 1273, 1226, 1165, 786, 756, 702 cm^−1^; ^1^H NMR (500 MHz, CDCl_3_) δ 8.12 (1H, d, *J* = 7.9 Hz), 8.07 (1H, d, *J* = 8.1 Hz), 7.69 (1H, t, *J* = 8.1 Hz), 7.53 (1H, t, *J* = 7.9 Hz), 4.68 (1H, dd, *J* = 8.3, 2.3 Hz), 3.93–3.89 (1H, m), 3.75–3.69 (1H, m), 3.06–3.02 (1H, m), 2.43–2.36 (1H, m), 2.29–2.21 (1H, m), 2.18–2.11 (1H, m); ^13^C NMR (125 MHz, CDCl_3_) δ 168.4, 163.8, 157.5, 146.4, 132.9, 132.5, 129.0, 128.5, 128.1, 121.4, 51.8, 47.9, 26.5, 23.3; MS (ES) *m*/*z* 314 [M + H]^+^; HRMS (ES): calculated for C_14_H_12_N_5_O_2_S [M + H]^+^ 314.0706, found 314.0699.

(2*S*)-6,14,16,20,21-Pentaazapentacyclo[12.7.0.0^2,6^.0^8,13^.0^15,20^] henicosa-1(21),8,10,12,15,17-hexaene-7,19-dione (**14**): To a 2.5-mL microwave vial were added Compound **12** (100 mg, 0.39 mmol) and ethyl propiolate (0.04 mg, 0.41 mmol) in EtOH (1 mL) with a stir bar. The reaction vessel was sealed and heated under microwave irradiation for 20 min at 150 °C. After cooling, the reaction vessel was uncapped, the vial contents poured into ice cold water, and the resulting precipitated solid was collected by filtration, washed with cold water and dried to give pure **14** (80 mg, 66%) as an off-white sold. mp 300 °C (decomp.); ν_max_ (KBr) 3093, 2947, 2885, 1643, 1519, 1473, 1411, 1087, 825, 763, 709 cm^−1^; ^1^H NMR (500 MHz, CDCl_3_) δ 8.21 (1H, d, *J* = 7.8 Hz), 8.07 (1H, d, *J* = 7.9 Hz), 7.93 (1H, t, *J* = 7.9 Hz), 7.69 (1H, t, *J* = 7.5 Hz), 7.54 (1H, t, *J* = 7.5 Hz), 6.39 (1H, d, *J* = 7.7 Hz), 4.73 (1H, m), 3.92–3.88 (1H, m), 3.75–3.70 (1H, m), 2.97–2.94 (1H, m), 2.48–2.40 (1H, m), 2.22–2.17 (2H, m); ^13^C NMR (125 MHz, CDCl_3_) δ 169.4, 163.9, 151.6, 149.1, 133.3, 132.7, 131.8, 128.9, 128.9, 128.5, 124.3, 112.8, 51.1, 47.6, 26.5, 23.4; MS (ES) *m*/*z* 308 [M + H]^+^; HRMS (ES): calculated for C_16_H_14_N_5_O_2_ [M + H]^+^ 308.1142, found 308.1141.

(2*S*)-17-Methyl-6,14,16,20,21-pentaazapentacyclo[12.7.0.0^2,6^.0^8,13^.0^15,20^] henicosa-1(21),8,10,12,15,17-hexaene-7,19-dione (**15**): To a 2.5-mL microwave vial were added Compound **12** (100 mg, 0.39 mmol), ethyl acetoacetate (56 mg, 0.43 mmol) and glacial acetic acid (1 mL) with a stir bar. The reaction vessel was sealed and heated under microwave irradiation for 20 min at 150 °C. After cooling, the reaction vessel was uncapped, the reaction mixture was concentrated and the residue suspended with ice cold water, and the resulting precipitated solid was collected by filtration, washed with cold water and dried to give pure **15** (90 mg, 72%) as an off-white solid. mp > 300 °C; ν_max_ (KBr) 3070, 2924, 2854, 1697, 1635, 1612, 1573, 1535, 1465, 1411, 1273, 1188, 1002, 887, 794, 756 cm^−1^; ^1^H NMR (500 MHz, CDCl_3_) δ 8.14 (1H, d, *J* = 8.2 Hz), 8.11 (1H, d, *J* = 7.9 Hz), 7.73 (1H, t, *J* = 7.9 Hz), 7.58 (1H, t, *J* = 7.9 Hz), 6.22 (1H, s), 4.77 (1H, dd, *J* = 8.3, 2.6 Hz), 3.90–3.85 (1H, m), 3.75–3.69 (1H, m), 3.27–3.22 (1H, m), 2.48–2.40 (1H, m), 2.39 (3H, s), 2.34–2.28 (1H, m), 2.18–2.11 (1H, m); ^13^C NMR (125 MHz, CDCl_3_) δ 164.7, 163.8, 156.4, 151.8, 147.9, 132.4, 131.9, 129.4, 129.1, 128.9, 123.8, 104.7, 51.4, 47.7, 26.6, 24.4, 23.5; MS (ES) *m*/*z* 322 [M + H]^+^; HRMS (ES): calculated for C_17_H_16_N_5_O_2_ [M + H]^+^ 322.1299, found 322.1297.

Ethyl (2*S*)-7,19-dioxo-6,14,16,20,21-pentaazapentacyclo [12.7.0.0^2,6^.0^8,13^.0^15,20^] henicosa-1(21),8,10,12,15,17-hexaene-18-carboxylate (**16**): To a 2.5-mL microwave vial were added Compound **12** (100 mg, 0.39 mmol), diethyl ethoxymethylenemalonate (93 mg, 043 mmol) and EtOH (2 mL) with a stir bar. The reaction vessel was sealed and heated under microwave irradiation for 20 min at 150 °C. After cooling, the reaction vessel was uncapped, the vial contents poured into of ice cold water, and the resulting precipitated solid was collected by filtration, washed with cold water and dried to give pure **16** (90 mg, 61%) as an off-white solid. mp 298–300 °C; ν_max_ (KBr) 2978, 1720, 1643, 1581, 1512, 1411, 1296, 1126, 794 cm^−1^; ^1^H NMR (500 MHz, CDCl_3_) δ 8.84 (1H, s), 8.14 (1H, d, *J* = 7.9 Hz), 8.08 (1H, d, *J* = 8.1 Hz), 7.76 (1H, t, *J* = 8.0 Hz), 7.63 (1H, t, *J* = 7.4 Hz), 4.81 (1H, dd, *J* = 8.3, 2.6 Hz), 4.40 (2H, q), 3.91–3.87 (1H, m), 3.76–3.70 (1H, m), 3.28–3.23 (1H, m), 2.52–2.46 (1H, m), 2.33–2.25 (1H, m), 2.20–2.13 (1H, m), 1.40 (3H, t, *J* = 7.1 Hz); ^13^C NMR (125 MHz, CDCl_3_) δ 164.1, 163.5, 159.5, 152.9, 152.5, 150.2, 132.5, 132.2, 129.6, 129.5, 128.4, 123.9, 109.3, 61.1, 51.4, 47.8, 26.6, 23.4, 14.2; MS (ES) *m*/*z* 380 [M + H]^+^; HRMS (ES): calculated for C_19_H_17_N_5_O_4_Na [M + Na]^+^ 402.1173, found 402.1177.

### 3.2. Animals

Male ICR mice, weighing 20–25 g, were used for the anticonvulsant, hypnotic and anxiolytic assays. All mice were used in accordance to the Guiding Principles and the experimental protocol (No. 102-250-B) was approved on 20 July 2013 by the Institutional Animal Care and Use Committee (IACUC) of the China Medical University. They were housed for at least 1 week before starting the experiment with free access to standard food pellets (supplied and designed by Fwusow Industry Co. LTD., Taiwan) and tap water and housed in a regulated environment (23 ± 1 °C temperature and 60% humidity), wherein a 12:12 h light/dark cycle (light phase: 08:00–20:00 h) was maintained. PBDT derivatives were administered, and the anticonvulsant, hypnotic and anxiolytic assays were performed using the double-blind method.

### 3.3. Picrotoxin- or Strychnine-Induced Convulsion in Mice

In brief, clonic–tonic convulsion was induced by a subcutaneous (sc) injection of picrotoxin or intraperitoneal (ip) injection of strychnine. The mice were pretreated with PBDT derivatives (1 mg/kg, ip), diazepam (1 mg/kg, ip) or vehicle, 15 min before the injection of picrotoxin (10 mg/kg, sc) or strychnine (2 mg/kg, ip). After the picrotoxin or strychnine injection, mice were placed in the testing chamber. The latencies to myoclonic jerks and the duration from clonic to tonic convulsion were recorded [33].

### 3.4. Pentobarbital-Induced Hypnotic Model in Mice

In brief, the hypnotic model was induced by an intraperitoneal injection of sodium pentobarbital. The mice were pretreated with PBDT derivatives (1 mg/kg, ip), diazepam (1 mg/kg, ip) or vehicle, 15 min before the injection of sodium pentobarbital (30 mg/kg, ip). After the sodium pentobarbital injection, mice were placed in the testing chamber. The latency to the loss of righting reflex (induction time in seconds) and the time required to recover righting reflex or awakening (sleeping time in minutes) were recorded [33].

### 3.5. Elevated Plus Maze in Mice

The elevated plus maze is comprised of two open arms (30 × 5 × 0.25 cm) and two closed arms (30 × 5 × 15 cm) that extended from a common central platform (5 × 5 cm) that was elevated to a height of 50 cm above the floor level. Mice were given PBDT derivatives (1 mg/kg, ip), diazepam (1 mg/kg, ip) or vehicle 15 min before their placement on the elevated plus maze. In the experimental period, every precaution was taken to ensure that no external stimuli could evoke anxiety in the mice. After each test, the maze was carefully cleaned up with a wet tissue paper (10% ethanol solution) to eliminate the interference of the olfactory cues on the next mice. All tests were recorded by a video camera and an automated video tracking system device equipped with Etho Vision XT software (Noldus Information Technology, Leesburg, VA, USA). The number of entries and the time spent in the open and closed arms were recorded during a 5-min test period. The percentage of arm entries in each arm (open or closed arm entry × 100/total entries) and the percentage of time spent in each arm (time spent in open or closed × 100/time spent in both arms) were calculated for each mouse [33].

### 3.6. Statistical Analysis

All data were expressed as the mean ± SEM for each experimental group. Statistical analysis was performed by one-way analysis of variance (ANOVA) followed by Dunnett’s test. When the probability (*p*) was less than 0.05, the difference was considered significant. IBM SPSS Statistics 12.0 (SPSS Inc., Chicago, IL, USA) was used in this study.

## 4. Conclusions

We have synthesized four pentacyclic benzodiazepine derivatives (PBDTs **13**–**16**) from 3-amino triazolopyrrolo[2,1-*c*][1,4]benzodiazepin-8-one (**12**) via conventional thermal heating and microwave-assisted intramolecular cyclocondensation. The biological evaluation of these compounds revealed that PBDT **13** possessed the best anticonvulsant, sedative and anxiolytic effects. There was no significant difference in potency between PBDT **13** and diazepam in our tests, and the action mechanism of PBDT **13** could be similar to that of diazepam via the benzodiazepine receptors.
